# CA125 as a Potential Biomarker in Non-Malignant Serous Effusions: Diagnostic and Prognostic Considerations

**DOI:** 10.3390/jcm14124152

**Published:** 2025-06-11

**Authors:** Lavinia Alice Bălăceanu, Cristiana Grigore, Ion Dina, Cristian-Dorin Gurău, Mara Mădălina Mihai, Beatrice Bălăceanu-Gurău

**Affiliations:** 1 Department of Medical Semiology, “Sf. Ioan” Clinical Emergency Hospital, “Carol Davila” University of Medicine and Pharmacy, 020021 Bucharest, Romania; alice.balaceanu@umfcd.ro (L.A.B.); cristiana.draganescu@drd.umfcd.ro (C.G.); 2 Internal Medicine Clinic, “Sf. Ioan” Clinical Emergency Hospital, 042122 Bucharest, Romania; 3 Clinical Department of Gastroenterology, “Sf. Ioan” Clinical Emergency Hospital, 042122 Bucharest, Romania; 4 Orthopedics and Traumatology Clinic, Clinical Emergency Hospital, “Carol Davila” University of Medicine and Pharmacy, 020021 Bucharest, Romania; 5 Department of Oncologic Dermatology, “Elias” Emergency University Hospital, “Carol Davila” University of Medicine and Pharmacy, 020021 Bucharest, Romania; mara.mihai@umfcd.ro (M.M.M.); beatrice.balaceanu@drd.umfcd.ro (B.B.-G.); 6 Research Institute of the University of Bucharest, University of Bucharest, 050663 Bucharest, Romania

**Keywords:** CA125, serous effusions, non-malignant ascitis, biomarkers, heart failure

## Abstract

**Background/Objectives:** Carbohydrate antigen 125 (CA125) is a glycoprotein commonly overexpressed in epithelial ovarian cancer and widely recognized as a tumor marker. However, elevated CA125 levels are also observed in various non-malignant conditions, including diseases affecting mucosal surfaces, pleural or peritoneal effusions, cirrhosis (with or without ascites), endometriosis, uterine fibroids, adenomyosis, pelvic inflammatory disease, and pregnancy. This review aims to explore the role of CA125 in non-malignant serous effusions, highlighting its diagnostic and prognostic potential beyond the realm of oncology. **Methods:** A comprehensive literature search was conducted across multiple databases and clinical trial registries. Eligible studies included full-text original research articles, reviews, and case reports published in English over the past 10 years. Inclusion criteria were limited to studies involving human subjects and focused on the role of CA125 in non-malignant serous effusions. **Results:** CA125 is produced by coelomic epithelial cells lining the ovary, pleura, pericardium, and peritoneum. Its serum concentration is not significantly influenced by age, body weight, or renal function, even in the advanced stages of the disease. In peritoneal conditions, CA125 is synthesized by mesothelial cells and serves as a potential marker of peritoneal involvement. The prevailing pathophysiological mechanism suggests that mechanical stretching of mesothelial cells due to ascitic pressure stimulates CA125 release. Similarly, in heart failure, mesothelial cells of the pericardium produce CA125, which correlates with congestion severity, supports risk stratification, and may inform diuretic therapy. **Conclusions:** While a threshold of 35 U/mL is established for malignancy, no standardized cutoff exists for CA125 in non-malignant conditions. The utility of CA125 measurement in peritoneal, pleural, or pericardial effusions—and cardiovascular diseases such as acute heart failure—for purposes of differential diagnosis, treatment guidance, or prognostication warrants further investigation through prospective clinical trials.

## 1. Introduction

Carbohydrate antigen 125 (CA125) is a high-molecular-weight glycoprotein most commonly associated with epithelial ovarian cancer, where it is frequently overexpressed and serves as a well-established tumor marker [1]. CA125 is the extracellular fragment of MUC16, a type I transmembrane mucin encoded by the MUC16 gene, notable for its extensive cDNA sequence and substantial molecular size [1,2]. Due to its large molecular structure, the intact MUC16 protein is not readily detectable in the serum; instead, serum assays typically measure its cleaved extracellular domain—recognized as CA125 [1]. The serum concentration of CA125 is influenced by its clearance through the hepatic reticuloendothelial system, and false elevations may occur due to antibody cross-reactivity with other circulating proteins [2].

While CA125 has long been established as a tumor marker in ovarian malignancy, its utility extends into broader gynecologic applications [3]. Elevated levels have also been reported in a variety of other malignancies, including breast, endometrial, lung, gastric, colorectal, pancreatic, and hepatocellular cancers, as well as in rarer entities such as Epstein-Barr virus-associated lymphoepithelioma-like cholangiocarcinoma and melanoma [2,4,5,6]. Hematologic malignancies, such as lymphoma and multiple myeloma, may also lead to increased CA125 levels in women [7]. Interestingly, Xiao et al. found that CA125 levels may not correlate with tumor size or peritoneal carcinomatosis in the absence of ascites [8]. Furthermore, simultaneous elevation of CA125 in both serum and ascitic fluid does not reliably differentiate between malignant and benign ascites, and the level of CA125 in ascitic fluid cannot distinguish between ovarian cancer-related ascites and those from other malignant or benign causes [9,10,11]. In gastric cancer, Feng et al. demonstrated that while serum CA125 is rarely elevated in early disease, its presence in peritoneal fluid is associated with peritoneal dissemination and poor prognosis [12]. These findings suggest that CA125 may aid clinical decision-making beyond adnexal masses, reinforcing the need to contextualize elevated levels within a broad diagnostic framework [3].

The diagnostic utility of CA125 in pleural effusions is similarly limited. Several studies, including those by Volarić et al. and Zhang et al., concluded that CA125 levels in pleural fluid do not significantly differ from serum levels and, thus, offer limited value in differentiating malignant from benign pleurisy [13,14]. The use of CA125, alone or in combination with other tumor markers, is not currently recommended in clinical algorithms for evaluating malignant pleural effusions due to insufficient diagnostic accuracy and high resource consumption [14].

Beyond oncology, CA125 is elevated in numerous non-malignant conditions, particularly those involving serosal inflammation or mechanical irritation. These include diseases affecting mucosal surfaces, pleural or peritoneal effusions, cirrhosis (with or without ascites), endometriosis, uterine fibroids, adenomyosis, pelvic inflammatory disease, and pregnancy [2,4]. Autoimmune diseases such as systemic lupus erythematosus, rheumatoid arthritis with interstitial lung involvement, and idiopathic pulmonary fibrosis have also been associated with elevated CA125 levels [2,4]. Hu et al. reported a strong correlation between systemic inflammation—reflected by elevated C-reactive protein—and increased CA125 concentrations, especially in postmenopausal women without evidence of malignancy [15]. Similarly, Gronnier et al. emphasized that in the context of marked inflammation, elevated CA125 cannot reliably discriminate between benign and malignant conditions [16]. Consequently, normal tumor marker levels in conjunction with negative imaging are considered reassuring, reducing the likelihood of an underlying malignancy [16]. Givens et al. recommend serial ultrasound monitoring every 4–6 weeks for up to 12 weeks in women presenting with an adnexal mass smaller than 10 cm and a CA125 level below 35 U/mL, regardless of menopausal status [17].

CA125 expanding clinical relevance, coupled with a lack of consistent guidelines on its interpretation outside oncology, highlights the need for a comprehensive review. The scientific rationale for this work lies in the observed yet underexplored utility of CA125 in non-malignant settings. Therefore, the objective of this review is to critically examine and synthesize current evidence on the role of CA125 in non-malignant serous effusions—including ascites, pleural, and pericardial fluids—with a focus on underlying mechanisms, clinical implications, and potential integration into diagnostic and therapeutic algorithms.

## 2. Materials and Methods

A comprehensive literature search was conducted to identify relevant studies examining the role of CA125 in non-malignant serous effusions, including peritoneal, pericardial, and pleural fluids. The search strategy encompassed multiple databases and registries—Web of Science Core Collection, PubMed, Elsevier ScienceDirect, Cochrane Database, and Google Scholar—as well as the websites of international clinical guidelines. The objective was to capture a broad spectrum of full-text original articles, systematic reviews, and case reports addressing CA125 in the context of non-malignant serosal involvement.

The initial search was conducted in November 2024, with monthly updates through April 2025. To ensure that the findings reflected current clinical evidence, only studies published within the last decade (from 1 January 2014 to 17 April 2025) were included.

The search strategy employed both Medical Subject Headings (MeSH) and free-text keywords to maximize sensitivity and relevance. Key terms included “carbohydrate antigen 125”, “CA125”, “CA-125”, “MUC16 protein”, “serous effusion”, “non-malignant ascites”, and “non-malignant.” These terms were carefully selected to ensure comprehensive retrieval while maintaining a specific focus on CA125 in non-malignant effusions.

**Figure 1:**
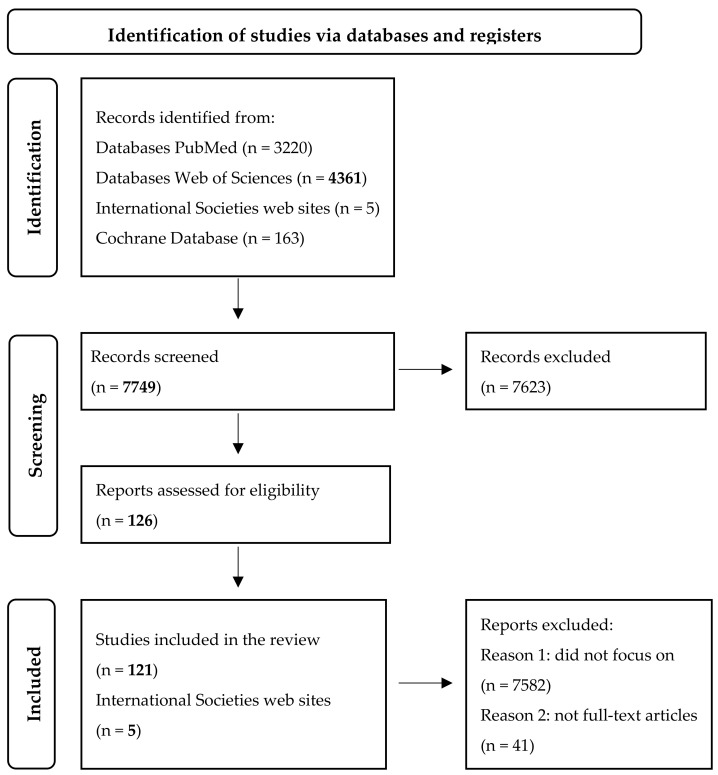
The flowchart underlines the studies included in our research [18].

To ensure methodological rigor and relevance, studies were screened using predefined inclusion and exclusion criteria. Eligible studies were limited to full-text original articles, reviews, and case reports published in English, involving human subjects, and specifically addressing CA125 in non-malignant serous effusions. Studies were excluded if they failed to address CA125 in the context of non-malignant ascites, pericardial involvement, or acute decompensated heart failure (Figure 1). Abstracts, non–peer-reviewed materials, conference proceedings, non-human studies, and articles without full-text availability were also excluded.

Data extraction was carried out systematically using a predefined rationale tailored for this review. The objective was to extract consistent and comprehensive information across the selected literature. Each study was assessed based on the clarity of its research objective, the robustness of its methodology, and the adequacy of its data synthesis approach. Extracted data were organized and tabulated using Microsoft Excel (Version 16.78.3, Microsoft Corporation, Redmond, WA, USA) to facilitate comparative analysis and synthesis.

A formal risk-of-bias assessment was not conducted due to the narrative design of this review. However, to minimize potential bias, we included only peer-reviewed, full-text studies published in English within the last decade and assessed each source for methodological clarity and relevance during data extraction.

## 3. Results

Trapé et al. reported that among non-malignant conditions, the most significant elevations in CA125 levels—exceeding 10-fold the upper reference limit—are observed in endometriosis, gynecological disorders, serous effusions, and cardiovascular diseases [9]. While some authors have shown that CA125 concentrations are unaffected by patient age, body weight, or renal function, others suggest that obesity is associated with lower serum CA125 levels due to increased plasma volume and hemodilution [19,20,21]. A comparative summary of key studies evaluating CA125 in non-malignant conditions is provided in Table 1.

### Pathophysiological Mechanisms of CA125 Synthesis

The physiological role of CA125 encompasses a wide array of processes, though its pathophysiological mechanisms remain incompletely elucidated. It is proposed to function as a lubricant for serous epithelia, offering mechanical protection, and is also involved in cellular and fluid transport, modulation of the immune response, inflammatory cascades, tumor cell dissemination, and tissue repair [29].

CA125 is synthesized by coelomic epithelial cells, including those lining the ovary, pleura, pericardium, and peritoneum [29,30]. In the peritoneum, it serves as a surrogate marker of serosal involvement [31]. A widely accepted mechanism involves the mechanical stretch of peritoneal mesothelial cells induced by ascitic fluid accumulation, which stimulates CA125 production and release [10,21,24,25]. This hypothesis is supported by findings that CA125 levels correlate only with ascites and not with esophageal varices or hepatorenal syndrome [25]. Edula et al. attribute elevated CA125 to lymphatic reabsorption of ascites and reduced hepatic clearance due to liver dysfunction [25]. Following large-volume paracentesis, CA125 levels decline significantly [23,24].

In liver cirrhosis, portal hypertension and splanchnic vasodilation trigger neurohormonal activation, renal vasoconstriction, fluid retention, and progressive renal impairment [32]. The onset of hepatorenal syndrome often coincides with cirrhotic cardiomyopathy, characterized by diastolic dysfunction, electrophysiological disturbances, and impaired contractile response [32]. In Budd–Chiari syndrome, elevated CA125 correlates with liver damage, ascites volume, Rotterdam score, recurrence risk, and overall prognosis, prompting Cheng et al. to advocate for dynamic monitoring of CA125 at admission, discharge, and follow-up [24].

de la Espriella et al. confirmed that CA125 levels remain stable even in severe renal impairment, including creatinine clearance below 30 mL/min/1.73 m*^2^* [33]. Fluid overload is linked to elevated serum CA125 levels not only in heart failure but also in chronic kidney disease and patients undergoing peritoneal dialysis [34]. High serum CA125 values predict poor ultrafiltration outcomes and the need for transition to hemodialysis [34]. Oliveira Júnior et al. proposed that serial measurements of CA125 in dialysate could serve as a biomarker for peritoneal inflammation and structural integrity, with implications for peritoneal fibrosis and dialysis efficiency [26].

Produced by mesothelial cells, including those of the pericardium, CA125 has emerged as a biomarker in heart failure for assessing congestion severity, guiding diuretic therapy, and stratifying risk [29,35]. By interacting with N-glycans, CA125 modulates galectin activity and extracellular matrix remodeling, potentially contributing to cardiac structural changes [22,29,35]. The underlying mechanism appears to involve mesothelial shear stress [11]. Núñez-Marín et al. proposed two mechanisms linking CA125 to intrarenal venous congestion in acute heart failure: systemic venous congestion resulting in increased hydrostatic pressure, and subclinical ascites causing elevated intra-abdominal pressure [36]. Hung et al. further suggested that left atrial dilation could mechanically stretch the pericardium, thereby increasing CA125 [28]. Kumric et al. associated CA125 levels with right atrial and pulmonary capillary pressures [37].

Elevated CA125 is more indicative of systemic venous congestion and right-sided heart dysfunction, whereas NT-proBNP correlates with left ventricular dysfunction, euvolemic status, or mild congestion [37]. CA125 elevation has also been linked to increased intra-abdominal pressure, mechanical mesothelial stress, and inflammation [38]. Proinflammatory cytokines such as TNF-α, IL-1, IL-10, and bacterial lipopolysaccharides contribute to CA125 upregulation [37,39,40]. Intestinal congestion in acute heart failure facilitates bacterial translocation and endotoxin production, further enhancing CA125 synthesis [40]. CA125 can also be elevated in pleural fluid irrespective of etiology [11].

Although a threshold of 35 U/mL is standard for malignancy, no specific cutoff has been established for non-malignant conditions such as heart failure [35]. In non-malignant effusions, CA125 levels typically rise 5–6 times above the upper limit of normal, but in some cases, they may reach levels up to 100-fold higher [9]. In cirrhosis without ascites, only 10% of patients exceed the reference range, and values rarely surpass 10-fold the upper limit [9]. Notably, in Budd–Chiari syndrome, CA125 levels do not significantly differ based on the presence or absence of hepatocellular carcinoma [24].

Large, gradually accumulating serous effusions are more likely to be associated with substantial CA125 elevations than rapid-onset effusions [35]. Beyond indicating fluid overload severity, CA125 provides insight into the chronicity of congestion, diuretic responsiveness, and prognosis [35]. Unlike NT-proBNP, CA125 levels are unaffected by age, renal function, or left ventricular parameters, making it potentially useful in evaluating fluid overload in elderly patients, those with cardiorenal syndrome, or those with preserved ejection fraction [35,41]. Serial CA125 measurements may assist in monitoring systemic venous congestion, particularly in right-sided heart failure [22].

CA125 has a biological half-life of 5–10 days [42]. Given its long half-life, serial monitoring over weeks or months may help assess the clinical course following decompensation [22]. In the BIOSTAT-CHF study, elevated CA125 was significantly associated with heart failure-related hospitalizations and 1-year all-cause mortality risk [43]. Other studies support the association of CA125 with severe congestion through pathways involving mechanical stress, inflammation, and endothelial dysfunction [44]. In cirrhosis with ascites, Zuckerman et al. reported a mean serum CA125 of 321 U/mL, with ascitic fluid levels exceeding those in serum [23]. Cheng et al. found that in Budd–Chiari syndrome, CA125 concentrations were significantly associated with ascites volume [24].

## 4. Discussion

### 4.1. CA125 in the Field of Gynecology

CA125 is predominantly recognized as a biomarker for ovarian neoplasms, especially for epithelial ovarian cancer. It was approved by the FDA in patients with epithelial ovarian cancer for monitoring treatment response [45]. It is also elevated in endometrial cancer [45].

In the early stages of epithelial ovarian cancer, CA125 levels are elevated in up to 50% of patients, the marker being specific to advanced stages of the disease, with elevated levels in about 85% of patients [46]. Serous ovarian cancers have a CA125 level of more than 300 U/mL [47]. CA125 is not so useful in other epithelial ovarian cancers or non-epithelial, early-stage, or premenopausal conditions [47]. In non-epithelial ovarian cancers, preoperative measurement of serum CA125 levels is recommended both for diagnosis and to monitor response to chemotherapy [48]. Combined models with multiple biomarkers associated with CA125 are reported by various authors with high specificity and sensitivity for ovarian cancer in postmenopausal patients [49,50,51,52,53,54,55].

Recent investigations have broadened CA125 diagnostic applicability to encompass various gynecological disorders, thereby underscoring its potential—albeit with certain constraints—in discerning between non-malignant conditions such as leiomyomas and adenomyosis, and malignant entities like endometrial carcinoma.

In detecting endometrial cancer among patients presenting with abnormal uterine bleeding, Nithin et al. found that CA125 alone had a sensitivity of 52.63% and specificity of 80%, emphasizing its utility as a non-invasive diagnostic tool [56]. Shawn LyBarger et al. showed that elevated CA125 levels were associated with advanced stage, lymphovascular space invasion, and lymph node metastasis in endometrial cancer patients, indicating its prognostic value [57].

The diagnostic specificity of CA125 for uterine leiomyosarcomas remains suboptimal, as elevations in serum levels may also manifest in a multitude of benign gynecological conditions and various other malignancies [3,58,59,60]. While some research studies suggest that preoperative elevations of CA125 may aid in distinguishing leiomyomas from leiomyosarcomas, other studies underscore the biomarker’s restricted diagnostic reliability due to significant overlaps in values between benign and malignant conditions [3,58,59]. Prognostic assessments have similarly reported inconsistent findings; while some data correlate elevated CA125 levels with advanced disease stage, unfavorable outcomes, or heightened recurrence risk, its status as an independent prognostic marker remains inadequately defined [3,61,62,63]. Immunohistochemical evaluations further complicate interpretation, demonstrating that while serum CA125 levels may be increased in certain uterine sarcomas, its expression within neoplastic tissue is often absent, thereby raising questions regarding the mesothelial cells origin of the elevated serum concentrations [3,64]. Even though certain evidence indicates that elevated CA125 levels may be associated with more aggressive tumor behavior in leiomyosarcoma, reliance solely on this biomarker for diagnostic purposes remains insufficient [3,61].

Regarding the guidelines, we do not have recommendations regarding the usefulness of preoperative CA125 determination or using tumor markers CA125 in combination or not with HE4 as the sole method in the differential diagnosis of benign/borderline or malignant ovarian tumors in the absence of imaging [65,66,67]. Determining serum levels of CA 19-9 and CEA, in addition to CA125 levels, can help in the differential diagnosis of gastrointestinal metastases from primary mucinous ovarian tumors [67].

CA125 levels are also elevated in other gynecologic non-malignant conditions such as adenomyosis, endometriosis, Meig tumor, uterine leiomyoma, pelvic inflammatory disease, or ovarian hyperstimulation [67]. The normal value of CA125 is 35 units/mL, but the cut-off is clinically arbitrary, mainly in premenopausal women [67]. The two assays used in clinical practice have different normal limits and a comparison is made using the same type of test [67]. CA125 level physiological varies with body mass index, smoking, and menstruation [67].

Kicheol et al. demonstrated that mean serum CA125 levels were significantly higher in women with adenomyosis compared to those with uterine fibroids, highlighting its potential in differential diagnosis [45]. Babacan et al. found that CA125 levels are influenced by tumor size and the presence of adenomyosis in patients with uterine fibroids, suggesting that elevated CA125 may not be specific to malignancy [68].

In adolescent girls with endometriosis, determining the CA125 level has not been proven useful either in diagnosing endometriosis or in correlating it with the type or severity of pain [69]. Other authors consider that CA125 has low sensitivity and variable specificity in endometriosis, although it correlates with the severity of the disease [70]. The combined determination of CA125 and HE4 is useful in the differential diagnosis of epithelial ovarian cancer from endometriotic ovarian cysts [71,72].

### 4.2. CA125 in Congestive Heart Failure

Congestion is a prevalent feature in chronic heart failure, affecting approximately 45% of outpatients, as reported in the CARDIOREN registry [73]. Gayán Ordás et al. proposed the combined use of CA125 and natriuretic peptides as complementary biomarkers for the early identification and management of heart failure exacerbations [73]. While the conventional cut-off value for CA125 remains 35 U/mL, Núñez et al. suggest that individualized thresholds may offer prognostic value [74]. It is important to note that, although 35 U/mL is the established oncologic reference, lower cutoffs—such as <23 U/mL in heart failure—have been proposed in specific non-malignant settings for risk stratification purposes, though these are not yet internationally standardized [74]. Specifically, CA125 levels below 23 U/mL are associated with a reduced risk of adverse events one month post-hospitalization and lower readmission and mortality rates at six months [74]. Similarly, Shi et al. demonstrated a strong association between elevated CA125 levels and an increased risk of hospitalization for heart failure [75].

In acute heart failure, CA125 correlates with signs of systemic venous congestion, such as peripheral edema and pleural effusion, as well as with the severity of tricuspid regurgitation assessed by echocardiography [76]. Elevated CA125 levels are useful in guiding therapeutic strategies. In cases of extravascular congestion with high CA125, intensified diuretic therapy—including sodium-glucose cotransporter 2 inhibitors—is recommended [75]. In contrast, vasodilators play a more significant role in intravascular congestion, where aggressive diuresis may be less effective [43].

The CHANCE trial reported that maintaining CA125 levels below 35 U/mL through optimized diuretic and statin therapy reduced hospital readmission for heart failure, although mortality outcomes remained unaffected [77]. García-Blas et al. similarly proposed CA125-guided intensification of diuretics in patients with acute heart failure and type 1 cardiorenal syndrome, demonstrating improved renal function in patients with elevated CA125 at admission [78]. Intrarenal venous flow with a monophasic pattern, indicative of renal congestion and strongly associated with CA125 levels, is also predictive of hospitalization and cardiovascular mortality [79].

Beyond congestion assessment, CA125 serves as a prognostic biomarker in heart failure [80]. The EMPEROR trial highlighted its modest role in predicting outcomes in patients with reduced ejection fraction but found no significant prognostic utility in heart failure with preserved ejection fraction (HFpEF) [81]. In contrast, Pacho et al. reported that while CA125 had limited predictive value for 30-day and one-year rehospitalization, it remained an independent predictor of all-cause mortality at one year [82]. Menghoum et al. reinforced its prognostic role in HFpEF, where elevated CA125 correlated with advanced NYHA class, right ventricular dysfunction, reduced TAPSE, tricuspid regurgitation, hepatic congestion (AST/ALT > 2), and increased inferior vena cava diameter [83]. Hung et al. also noted that CA125 levels correlated with maximal left atrial volume and predicted hospitalization in women with HFpEF [28].

However, other studies have questioned the incremental value of CA125. Rubio-Gracia et al. found that when markers such as NT-proBNP and relative plasma volume were considered, CA125 did not significantly improve mortality prediction in elderly patients with acute decompensated heart failure [84]. Given CA125’s half-life of 5–10 days, serial measurement during hospitalization may enhance prognostic evaluation following two half-lives (approximately 10 days) [85]. In cases of acute heart failure with severely impaired renal function, CA125 may surpass NT-proBNP in predicting one-year survival [85]. Núñez-Marín et al. further identified CA125 as an independent marker for intrarenal venous congestion, with a non-linear relationship and a significant threshold at 63.5 U/mL [36].

### 4.3. CA125 in Other Cardiac Pathologies

Beyond heart failure, CA125 elevation has been observed in other cardiac diseases. In dilated cardiomyopathy, Amorim et al. found correlations between CA125 levels and echocardiographic parameters including left atrial volume, E/A and E/e′ ratios, and pulmonary artery systolic pressure, along with inflammatory markers such as hs-CRP and uric acid [86]. In cardiac amyloidosis, elevated CA125 levels have been associated with polyserositis, advanced Mayo staging, and reduced survival [87]. Li et al. suggest that CA125 may be non-inferior to NT-proBNP, troponin, and LDH in predicting outcomes in light-chain amyloidosis, while Wu et al. linked CA125 with cardiorenal amyloidosis progression [87,88].

CA125 also has prognostic value in patients undergoing surgical ventricular restoration. Kang et al. and Nan et al. proposed that elevated preoperative CA125 levels—specifically, above 13.825 U/mL—are associated with worse outcomes in patients with left ventricular aneurysms undergoing surgical reconstruction [89,90]. In ST-elevation myocardial infarction (STEMI), Falcão et al. identified a CA125 cut-off of 12.45 U/mL with prognostic accuracy comparable to NT-proBNP based on Killip classification, and Yndigegn et al. confirmed that CA125 predicts six-week mortality, as well as left ventricular dysfunction and remodeling at one year post-ACS [91,92]. These findings suggest that CA125 may reflect myocardial ischemia-induced serosal stress and systemic inflammation, contributing to long-term cardiac remodeling and heart failure [92,93].

Additionally, CA125 may serve as a screening biomarker for atrial fibrillation (AF). Sekiguchi et al. found that levels above 9.8 U/mL were predictive of new-onset AF in postmenopausal women, while Arbault-Biton et al. demonstrated that CA125 correlated with CHA2DS2-VASc scores and could detect AF of <48 h duration as accurately as NT-proBNP [94,95]. The duration of AF has also been shown to influence CA125 levels independently of heart rate, renal function, and dyspnea [95,96].

### 4.4. CA125 in End-Stage Liver Disease

CA125 is frequently elevated in advanced liver disease, even in the absence of malignancy [97,98]. Elevated levels are observed in both serum and ascitic fluid, with the latter often exceeding serum concentrations [11,23]. Zuckermann et al. noted a rapid decrease in CA125 following large-volume paracentesis, supporting a direct relationship between ascitic burden and CA125 levels [23]. Collazos et al. found that 98.4% of cirrhotic patients with ascites had elevated serum CA125, compared to only 4.1% of cirrhotics without ascites [99]. Qureshi et al. and Bergmann et al. confirmed that CA125 correlates more strongly with ascite volume than with liver function [100,101].

In cirrhosis, peritoneal mesothelial cells appear to be the primary source of CA125 production [102]. Aguilar-Reina et al. suggested that CA125 may help predict ascite recurrence in cirrhotic patients, and Trapé et al. reported that the ascitic fluid/serum CA125 ratio often exceeds 1 in benign effusions of hepatic or cardiac origin [103,104]. Devarbhavi et al. showed that ascites, not etiology, determine CA125 levels, and found no significant changes in CA125 one month after starting peritoneal dialysis [105]. In liver transplant candidates, CA125 correlates with MELD score, ascites, and alcoholic liver disease, and is most elevated in hepatitis B and cryptogenic liver failure [106,107]. Impaired protein clearance may be a key mechanism of CA125 elevation in these patients [107].

### 4.5. CA125 in Autoimmune Diseases

In systemic lupus erythematosus (SLE), elevated CA125 levels are linked to active disease and serosal involvement. Zhong et al. demonstrated associations with pleurisy and ascites, characterizing pseudo-Meigs’ syndrome [27,108].

### 4.6. CA125 in Clinical Guidelines

Despite its emerging utility, CA125 is not yet incorporated into major international guidelines for managing non-malignant ascites in cirrhosis [109,110,111,112,113,114] (Table 1). European heart failure guidelines mentioned CA125 as a potentially useful biomarker in 2020, but it was omitted from the 2023 update, reflecting ongoing uncertainty about its routine clinical application [115,116]. The Japanese heart failure guideline mentions CA125 among other biomarkers for personalized management, considering the complex pathophysiological mechanisms of heart failure [117] (Table 2). ACC/AHA guidelines do not mention CA 125 in the management of heart failure [118,119]. International guidelines for systemic lupus erythematosus do not mention CA125 in management [120,121,122,123].

Further prospective studies and randomized trials are needed to validate the clinical value of CA125 in managing non-malignant serous effusions, particularly in stratifying congestion severity, guiding therapy, and predicting outcomes. Additionally, further exploration of the molecular and cellular mechanisms underlying CA125 elevation in non-malignant settings is warranted.

### 4.7. Strengths and Limitations of the Study

This review has several strengths. It addresses a clinically relevant but underexplored topic—namely, the behavior and implications of CA125 in non-malignant serous effusions. A structured literature search was performed across multiple databases, and studies from diverse medical domains were included, offering a multidisciplinary perspective on the biomarker’s diagnostic and prognostic roles. The review also provides a comparative tabular summary to enhance the accessibility and synthesis of the data.

However, several limitations should be acknowledged. First, this is a narrative review rather than a systematic review or meta-analysis; while efforts were made to ensure methodological rigor, formal risk-of-bias assessment tools were not applied. Second, the heterogeneity of included studies—varying in design, population size, endpoints, and clinical settings—limits the generalizability and precludes quantitative synthesis. Third, publication bias may have influenced the findings, as studies with negative or inconclusive results are less likely to be published or indexed. Finally, most data on CA125 in non-malignant conditions are derived from retrospective studies, case reports, or small cohorts, which may affect the strength of the evidence and limit definitive clinical recommendations.

### 4.8. Future Perspectives

Given the expanding interest in the diagnostic and prognostic relevance of CA125 beyond oncology, future research should prioritize prospective, multicenter studies to validate condition-specific cutoff values in non-malignant settings such as heart failure, cardiometabolic disorders, cirrhosis, and autoimmune diseases.

In the context of chronic heart failure, a significant association between elevated CA125 levels and increased all-cause mortality has been noted, particularly in patients with HFpEF [40,124,125]. Similarly, Bayes-Genis et al. highlight its value in risk stratification, congestion monitoring, and therapeutic guidance [29]. These findings support the potential for CA125 to be incorporated into multiparametric clinical algorithms.

Furthermore, prospective studies such as the BIOSTAT-CHF Study underscore CA125’s potential in differentiating intravascular versus extravascular congestion, while emerging hypotheses suggest that CA125 may even serve as a future therapeutic target in cardiometabolic disorders [43].

Edula et al. found elevated CA125 levels in 85% of cirrhotic patients, correlating closely with ascites burden and hepatic dysfunction [25]. This points to a possible role for CA125 in tracking decompensation severity and fluid accumulation in advanced liver disease [25].

In autoimmune diseases, elevated CA125 levels have been reported in association with pleuritis, pericarditis, or peritoneal involvement, reflecting serosal irritation and immune-mediated fluid accumulation [27,108,126]. Given that mesothelial cells are both mechanically and immunologically responsive, CA125 expression may reflect disease activity or flare severity [27,108,126]. Future research should focus on elucidating the immunopathological mechanisms linking mesothelial cell activation and CA125 secretion in autoimmune settings.

Integrating CA125 with other biomarkers, such as NT-proBNP, HE4, or inflammatory mediators, may enhance its specificity and clinical utility in complex cases [40]. In parallel, mechanistic studies exploring the molecular pathways underlying CA125 expression in serosal and mesothelial tissues could clarify its role in systemic inflammation and congestion [40]. Finally, efforts should be made to include CA125 in disease-specific clinical guidelines and biomarker panels once sufficient evidence supports its standardized use.

## 5. Conclusions

CA125, traditionally regarded as a tumor marker, has broader clinical relevance that extends beyond malignancy. Elevated CA125 levels are observed not only in various cancers but also in a wide range of non-malignant conditions. Importantly, serum CA125 concentrations are not significantly influenced by patient age, body weight, or renal function—even in advanced stages of organ dysfunction. While the standard threshold of 35 U/mL is established for malignancy, CA125 levels in non-malignant serous effusions typically rise to 5–6 times above this reference limit and, in certain cases, may exceed it by up to 100-fold. Despite these findings, there is currently no consensus on a specific cutoff value for CA125 in non-malignant conditions. However, condition-specific thresholds have been proposed in the literature—for example, levels below 23 U/mL have been associated with a favorable prognosis in chronic heart failure—though such cutoffs are not yet standardized or widely adopted.

The diagnostic, therapeutic, and prognostic utility of CA125 in non-malignant serous effusions—particularly those related to peritoneal, pleural, or pericardial involvement, as well as in acute and chronic heart failure—remains incompletely defined. Further clinical trials are needed to validate the role of CA125 as a biomarker for differential diagnosis, treatment monitoring, and risk stratification in these settings.

## Figures and Tables

**Table 1:** Summary of key studies on CA125 in non-malignant conditions.

Author (Year)	Study Design	Population	Condition	CA125 Context	Key Findings
Núñez et al. (2021) [22]	Review	Acute heart failure patients	Heart failure	Serum CA125 < 23 U/mL	Monitoring and treatment guidance
Zuckerman et al. (1999) [23]	Observational	Patients with cirrhotic ascites	Liver cirrhosis	Serum CA125: mean 321 U/mL	Higher ascitic CA125 than serum; related to fluid overload
Cheng et al. (2015) [24]	Prospective	Patients with Budd-Chiari syndrome	Budd–Chiari syndrome	Serum CA125 associated with ascites volume	Correlated with prognosis and recurrence risk
Edula et al. (2018) [25]	Retrospective	Cirrhosis with ascites	Liver cirrhosis	Serum CA125 linked to fluid volume	Correlated with a degree of decompensation
Oliveira Júnior et al. (2021) [26]	Observational	Patients on peritoneal dialysis	CKD/Peritoneal dialysis	CA125 in dialysate	Marker for peritoneal inflammation and fibrosis risk
Trapé et al. (2024) [9]	Review	Various non-malignant conditions	Endometriosis, gynecologic disease, effusions, cardiovascular disease	Serum CA125 > 10× normal in some benign states	Marked elevation is possible even in benign gynecologic cases
Zhong et al. (2024) [27]	Retrospective	Patients with systemic lupus erythematosus	SLE	Associated with pleurisy and ascites	Reflects serosal involvement in active disease
Hung et al. (2012) [28]	Prospective	Women with HFpEF	Heart failure with preserved EF	Linked to left atrial volume	Predictor of hospitalization and congestion severity

**Table 2:** Major clinical guidelines reviewed for references to CA125 in heart failure and cirrhosis.

Guidelines	Year	References
EASL	2018	[109]
AASLD	2021	[110]
CMA	2024	[111]
Austrian Consensus	2023	[112]
APASL	2019	[113]
NICE	2023	[114]
ESC	2021	[115]
ESC	2023	[116]
JCS/JHFS	2021	[117]
ACC/AHA	2022	[118]
ACC/AHA	2024	[119]
EULAR	2023	[120]
BSR	2018	[121]
CRA	2018	[122]
Asia-Pacific	2021	[123]

## Data Availability

This review summarizes data reported in the literature and it does not report primary data.

## Abbreviations

CA125	Carbohydrate antigen 125
CA 19-9	Carbohydrate antigen 19-9
CEA	Carcinoembryonic antigen
MUC16	Mucin16
TAPSE	Tricuspid annular plane systolic excursion
AST	Aspartate transaminase
ALT	Alanine transaminase
LDH	Lactate dehydrogenase
STEMI	ST-segment elevation myocardial infarction
BNP	B type natriuretic peptide
NTproBNP	N-terminal pro-B type natriuretic peptide
